# Dental Antimicrobial Stewardship: Developing a Mobile Application for Rational Antibiotic Prescribing to Tackle Misdiagnosis

**DOI:** 10.3390/antibiotics13121135

**Published:** 2024-11-26

**Authors:** Jelena Roganović, Stefan Djordjević, Milena Barać, Jasna Crnjanski, Ivana Milanović, Jugoslav Ilić

**Affiliations:** 1 Department of Pharmacology in Dentistry, Faculty of Dental Medicine, University of Belgrade, 11000 Belgrade, Serbia; milena.barac@stomf.bg.ac.rs; 2 IMP-Computer Systems Ltd. (IMP-CS), Institute Mihajlo Pupin, 11000 Belgrade, Serbia; stefan.djordjevic@pupin.rs; 3 School of Electrical Engineering, University of Belgrade, 11000 Belgrade, Serbia; jasna.crnjanski@etf.bg.ac.rs; 4 Department of Restorative Odontology and Endodontics, School of Dental Medicine, University of Belgrade, 11000 Belgrade, Serbia; ivana.milanovic@stomf.bg.ac.rs (I.M.); jugoslav.ilic@stomf.bg.ac.rs (J.I.)

**Keywords:** antimicrobial stewardship, mobile application, misdiagnosis, dentistry

## Abstract

Background/Objectives: Inexperienced dentists and dental students are especially prone to misdiagnosis, and this represents a huge problem regarding antimicrobial stewardship. We aimed to develop a mobile app for rational antibiotic prescribing in dentistry based on local–systemic symptoms and patient factors, rather than solely on diagnosis, to tackle misdiagnosis. Methods: The study involved 64 participants, 50 of which were third-year dental students attending a pharmacology course focusing on antimicrobials, comprising lectures and practical sessions without (noAPP group, n = 22) or with (APP group n = 28) the assistance of a mobile application. The other 14 participants were practicing dentists who decided to register and use the application. All registered users of the application were asked to take a feedback survey, while learning outcomes were evaluated via a pharmacology quiz. Results: A decision tree was used for application development. In total, 76 impressions were collected on the application. The majority of the impressions were related to odontogenic–endodontic infections. Multiple linear regression analysis did not reveal differences in survey responses between practicing dentists and undergraduate students in the feedback survey responses. There was a significant difference in the mean pharmacology test scores between the noAPP and APP groups (5.50 ± 1.80 vs. 7.21 ± 1.03, *p* = 0.0001). Conclusions: The dentalantibiotic.com application was developed to support rational antibiotic prescribing, in view of tackling misdiagnosis, among inexperienced dentists, as well as to assist in undergraduates’ pharmacology learning, and the current study shows its large impact as an educational tool. The majority of participants considered it easy to use, efficient in facilitating the right antibiotic choice, and useful for everyday decision-making.

## 1. Introduction

Diagnostic accuracy is essential for antimicrobial stewardship. A retrospective study by Filice and colleagues showed that among five hundred randomly selected inpatients on antimicrobials, 95% of patients received inappropriate antibiotics due to an incorrect or indeterminate diagnosis, while only 58% of patients received a correct diagnosis [1], indicating that diagnostic errors may represent a huge problem in rational antibiotic prescribing.

In dentistry, systemic antimicrobial therapy is often prescribed for infective endocarditis prophylaxis [2] or for the treatment of bacterial infections; it is solely used as an adjunct to mechanical treatment [3,4,5]. In the treatment of endodontic or odontogenic infections, the first-choice antibiotic is amoxicillin, used alone or in combination with metronidazole. However, if patients are penicillin-allergic, clindamycin or azithromycin is recommended [3,4,5]. Inappropriate prescription leads to long-term adverse effects, such as antibiotic resistance, dysbiosis, and an increased susceptibility to opportunistic infections, like fungal infection, as well as an enhanced likelihood of allergic reactions. Moreover, misdiagnosis is followed by clinical treatment failure, adverse drug effects, and avoidable financial costs [3,4]. The knowledge and skills of dental professionals are very well-documented factors that may lead to errors such as misdiagnosis. Likewise, different dentists may have differing opinions on the same case, leading to an unclear diagnosis [6]. Inexperienced dentists and dental students are especially prone to misdiagnosing patients since experience level, along with a related lack of communication with patients and not looking at patients systemically, is an important factor in terms of diagnostic errors [7,8].

In order to improve the use of antibiotics in dentistry, we aimed to develop an application that is easily accessible to students and inexperienced dental professionals to decrease unnecessary and potentially harmful antibiotic use due to orofacial pain misdiagnosis. Right from the start, it is of huge importance for students to learn to work in the best interest of the patient in terms of their overall health and not just the health of their teeth, and this learning can be facilitated by utilizing optimal educational strategies to improve students’ diagnostic ability.

Mobile technologies provide broad accessibility and may facilitate learning by creating an environment in which students are motivated to participate in group discussions and dialogues and can quickly receive feedback from teachers [9]. Currently, mobile applications for rational antibiotic prescribing are mainly found in the form of manuals or clinical guidelines, which rely on diagnosis-specific or drug-input-specific features; offer detailed drug information, including information regarding indications, dosages, and potential interactions; and may even integrate local resistance data to inform prescribing decisions. Dental apps are more like a reference for the commonly prescribed drugs (antibiotics) in dentistry (Dental Drugs^®^, Lexicomp app^®^, Epocrates^®^) or a drug list for proposed dental condition diagnoses (Dental Prescriber^®^, UpToDate^®^). Thus, we developed a mobile app for rational antibiotic prescribing based on local–systemic symptoms and patient factors rather than solely on diagnosis, aiming to help dental students and inexperienced dentists adopt the principle of using antibiotics only when truly needed and provide treatment tailored to individual patient needs.

## 2. Results

**Figure 1:**
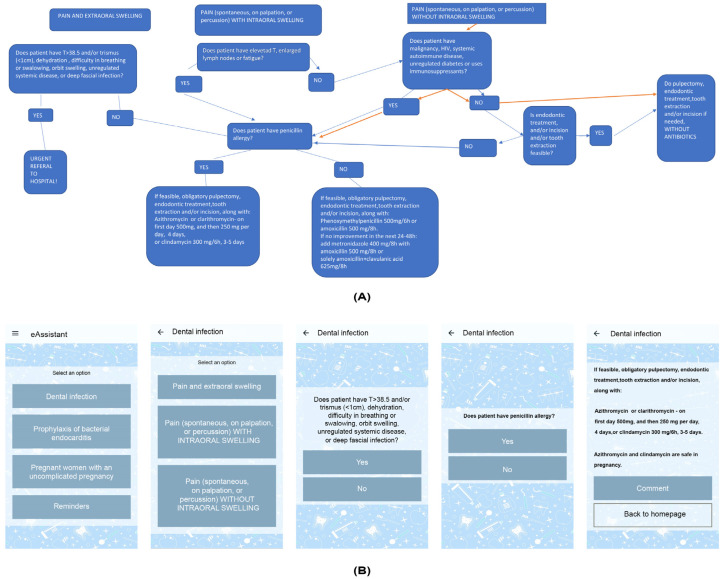
The decision tree (**A**) and the graphical interface of the application (https://www.dentalantibiotic.com/protocol; accessed on 4 November 2024) (**B**).

The decision tree structure was used as a guideline algorithm for the development of the application, enabling the establishment of a simple and clear interface, easy navigation, and quick access to prioritized content (Figure 1).

### 2.1. Feedback on Undergraduate Students’ and Practicing Dentists’ App Experience

**Figure 2:**
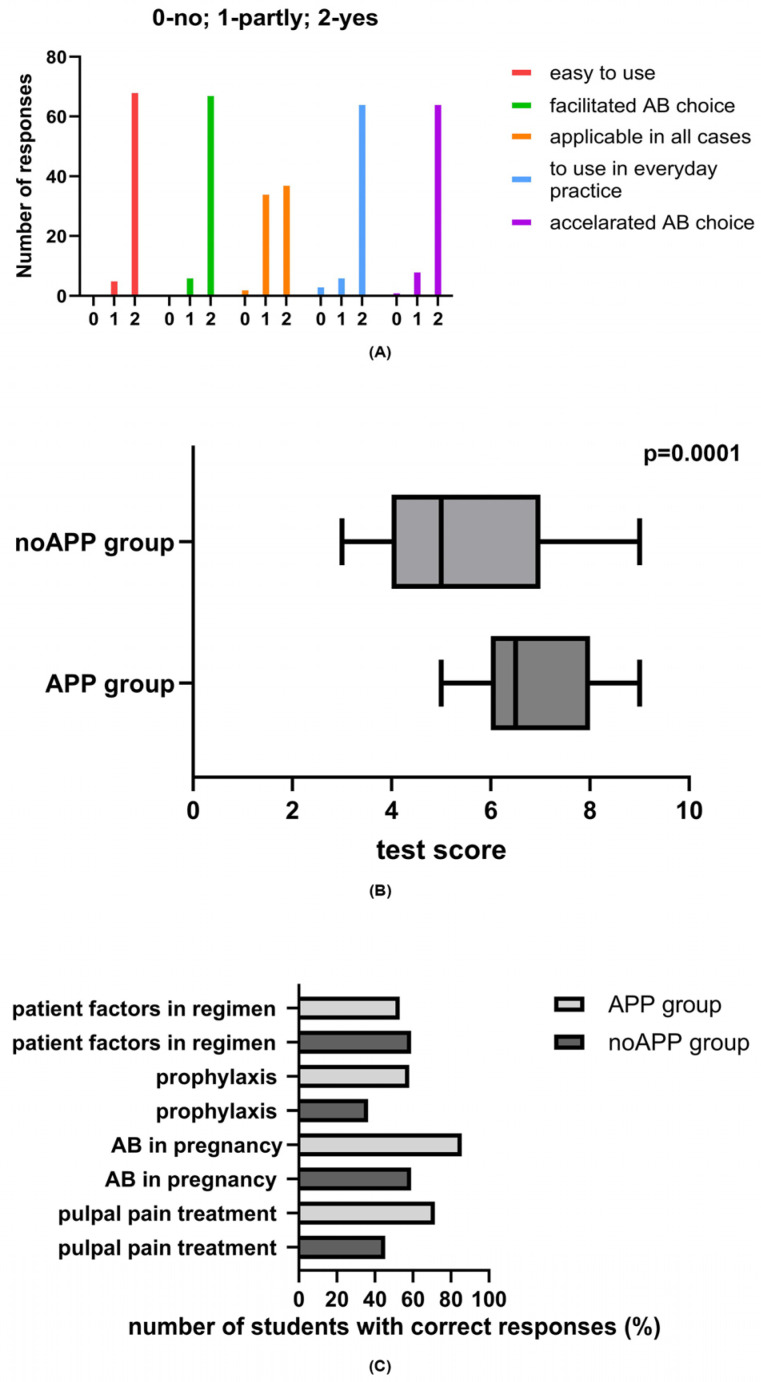
Distribution of responses to survey questions (**A**), as well as pharmacology test scores (**B**), and frequency of students with correct answers on proper pharmacological treatment of pulpal and periapical pain and swelling, endocarditis prophylaxis, antibiotic use in pregnancy, and antibiotic prescription-related patient factors (**C**) among students who used the application (APP group) and those who did not use the application (noAPP group).

In total, seventy-six impressions from sixty application users were collected: out of the fifty undergraduates, four did not respond; thirty responded once, and sixteen responded twice, while all practical dentists responded once. Moreover, four out of sixty provided incomplete responses, and the error rate was 6.6%. The majority of the impressions were related to odontogenic–endodontic infections (n = 44), followed by impressions taken for endocarditis prophylaxis treatment (n = 11), for ensuring proper antibiotic use in pregnancy (n = 11), and for important information (n = 10). The survey responses are presented in Figure 2A. The mean grade for assisting in learning was 4.67, while the value for everyday utility was 4.55. The students’ comments expressed satisfactory learning: *“The app enables easy access, systematicity and visibility in relation to syllabus”; “Simple, practical app, I am satisfied”*; *“A very useful app and easy to use. Ideal as a reminder for studying, and also during the prescribing antibiotics”*. The practicing dentists also found the application useful for everyday practice: *“The app is simple, fast and accurate. I think it is of great importance especially for young dentists who don’t have enough experience. It includes adults, pregnant women and patients at risk. However, I think it would be useful to include therapy in children, as well. We were not so familiar with prescribing drugs for children during the Pharmacology in Dentistry course, although the children are frequent patients in practice”*. Multiple linear regression analysis did not reveal differences in survey responses between the practicing dentists and undergraduate students in the feedback survey results (Table 1).

### 2.2. Pharmacology Test Scores

All fifty undergraduate students took the pharmacology test. There was a significant difference in the mean test scores between the noAPP and APP groups (5.50 ± 1.80 vs. 7.21 ± 1.03, *p* = 0.0001) (Figure 2B).

The difference observed is mainly reflected by the better scores regarding proper management of dental (pulpal) pain with/without swelling, as students who did not use application were wrong about prescribing antibiotics in the case of pulpal pain only and/or with intraoral swelling in immunocompetent patients instead of using dental treatments such as pulpectomy, nonsurgical root canal treatment, or incision and drainage for symptomatic irreversible pulpitis (the percentage of students with correct answers in the APP group was 71.5%, compared to 45.4% in the noAPP group). Another major stumbling block for students from the noAPP group was the endocarditis prophylaxis protocol, as the majority of the students prioritized amoxicillin and clavulanic acid instead of amoxicillin, as well as the use of erythromycin for patients with a penicillin allergy, while only 36.4% provided correct answers. However, when it comes to patient factors that influence rational antibiotic prescribing, such as allergy to penicillin and odontogenic condition progression to systemic involvement, there were no differences between students from the APP and noAPP groups (Figure 2C).

## 3. Discussion

In dental practice, professionals frequently make empiric decisions about antibiotic treatment. To make things more complex, symptoms may vary among patients with the same diagnosis, and different patients with the same diagnosis may require different medications or dosages based on their symptom profiles or comorbidities; thus, a personal treatment approach is recommended. Furthermore, significant differences often exist among clinicians in how they interpret clinical findings and radiographic data [10]. At the same time, third-year dental students are not familiar with orofacial pain differential diagnoses, and they are yet to develop the knowledge and skills needed to integrate an intraoral examination into their routine practice [11]. Thus, solely applying the usual educational approach, which relies on diagnosis-based antibiotic treatment, may not be fruitful.

Our application is based on an evidence-based decision tree structure, which uses a tree-like model of decisions and their possible implications: each node represents a “yes/no” question, while each branch represents the subsequent outcome. This is the first dental application for antibiotics prescribing based on such a concept. The tree structure is intuitive and easy to interpret [8], even for undergraduate third-year students which lack clinical knowledge, providing a straightforward method for making decisions (in clinical settings) based on yes/no questions. Recently, a decision tree model which assists clinicians in determining whether standard antibiotic empiric therapy is sufficient or therapy with broad-spectrum antibiotics is needed for children with Gram-negative bloodstream infections was developed [12]. Another mobile application named “Abx SteWARdS” was developed to provide physicians with clinical decision support regarding antibiotic prescribing for uncomplicated upper respiratory tract infections [13]. Likewise, a decision tree that stratifies COVID-19’s effects on biological systems was developed to build a stratified framework for a chatbot application [14]. The simplicity and utility of such an educational approach was highly valued by our study participants, including both undergraduate and practicing dentists. The majority of participants considered the application easy to use and efficient in facilitating the right antibiotic choice, also finding it useful for everyday decision-making.

The limitations of the current version of the application include the fact that recommendations do not include follow-up, local resistance patterns, or drug–drug interactions. The decision tree structure did not include contextual patient factors, such as socio-economic factors or patient preferences, which are also important in decision-making for antibiotic use. Moreover, the application is currently designed to be used only by dentists. However, the application could be upgraded in a way that allows for the creation of integrated interdisciplinary treatment plans, allowing other medical specialists to input their treatment goals and interventions, which can be shared and reviewed by the interdisciplinary team.

Nevertheless, the application recommendations took into account penicillin allergy and underlying medical conditions, and aim to prevent delay in providing antibiotics or urgent care for life-threatening situations, since urgent hospitalization for time-sensitive and severe infections are recommended. It is worth noting that dentists are advised to warn patients of the adverse effects associated with the use of specific antibiotics, which helps build trust between the doctor and patient and reduces anxiety because the patient knows what to expect and understands that the doctor has considered both the benefits and risks of the recommended antibiotic. Likewise, the decision tree is applicable to other populations of patients, such as pediatric patients; however, in such cases, recommendations may differ primarily in terms of antibiotic dosage, choice of antibiotic, and drug formulation.

Our application is based on American Dental Association (ADA) recommendations [2,5], is in accordance with guidelines used in the majority of European countries [3,4,15], and emphasizes avoidance of unnecessary antibiotic use with obligatory non-pharmacological causal treatment of orofacial pain. It should be noted that ADA protocols differ from other national protocols, such as the guidelines in countries like the UK (National Institute for Health and Care Excellence (NICE)), which are more restrictive in their recommendations for antibiotic prophylaxis. For instance, the NICE guidelines do not routinely recommend prophylactic antibiotics for patients with cardiac conditions undergoing dental procedures [16], although this approach has been challenged very recently [17]. Furthermore, the ADA recommends amoxicillin as the first choice for dental infections, while in parts of Europe, penicillin V is often preferred over amoxicillin for dental infections due to its more targeted bacterial action and lower impact on the microbiome, while cephalosporins are favored by dentists in Japan [15]. Notably, the ADA does not recommend clindamycin as an antibiotic of choice for penicillin allergy; however, we left it in our recommendations as a second choice, after azithromycin/clarithromycin, due to its good pharmacological properties [3], while warning the dentists to carefully monitor its use.

Proper antibiotic prescribing is critical to mitigate higher risk of mortality, complications, or secondary infections due to growing bacterial resistance and antibiotic ineffectiveness. Misdiagnosis and inappropriate prescribing can also lead to misinformed patient expectations [18]. Namely, if patients are unnecessarily prescribed with antibiotics, they may believe that antibiotics are always necessary for infections, leading to self-medication or causing them to always demand antibiotics always from their dentist, even when they are not needed. In line with this, our recent article showed that, despite being keen to follow evidence-based and updated guidelines, practicing dentists misuse antibiotics due to another neglected issue regarding antibiotic stewardship: the lack of ethics knowledge or disobedience of ethical principles when prescribing antibiotics [18]. Thus, we are of the opinion that undergraduate studies represent the ideal “place and time” to establish a pharmacological and ethical foundation essential for ensuring that, as students transition into independent dental practitioners—for whom financial considerations may become a primary focus—they maintain a commitment to patient safety and responsible antibiotic use. Therefore, our aim is to implement these application guidelines among undergraduate students to impose proper and ethical drug prescribing practices early, before the pressure of private practice can influence decision-making. The present application addresses several clinical problems related to antibiotic prescribing in dentistry by providing solutions that promote rational, evidence-based decisions; it provides clear guidelines on when antibiotics are truly necessary, minimizes unnecessary broad-spectrum antibiotic use, helps students and inexperienced dentists to assess the risks and benefits of prescribing antibiotics for each patient, considers patient-specific factors and antibiotic side effects, and prepares users for ethical decision-making in real-world practice, where profit may be a consideration.

When measuring the application’s utility in terms of learning, students who used the application showed much better pharmacology test scores, and in practical terms, the effect size of 1.20 indicates the large impact of the educational tool we were studying. The major effects of using the app were reflected in the improved inspection of symptoms requiring different management strategies, as these symptoms might be missed when focusing on diagnosis alone, as well as in the consideration of both symptom severity and patient history, allowing for a comprehensive evaluation of symptoms. By considering patient factors such as allergies, the application can help in reducing or avoiding adverse drug events [19]. Learning about drugs and symptoms encourages a deeper understanding of pharmacodynamics and pharmacokinetics, fostering mechanistic understanding, which has been shown to be useful; the students who used app rarely decided to use broad-spectrum antibiotics such as amoxicillin + clavulanic acid, which could drive resistance. Likewise, the majority of the APP group was cautious when considering the right antibiotic choice in pregnancy.

By focusing on symptoms and patient-specific factors (e.g., age, comorbidities, allergies, signs of systemic infection), the application can suggest more precise and effective antibiotic treatments tailored to individual patient needs [20]. Moreover, effective symptom management is crucial for patient wellbeing. Focusing solely on diagnoses may lead to the neglect of symptomatic treatments that can significantly improve patients’ quality of life. Practicing dentists did not differ from undergraduates in terms of satisfaction with the app. By integrating the latest clinical guidelines and providing simple, straightforward decision support, our application can help practicing dentists in delivering high-quality, evidence-based care and ensuring antibiotic stewardship.

## 4. Materials and Methods

### 4.1. Participants and Study Design

This study was approved by the Ethical committee of the School of Dental medicine (approval number: 36/51 2023) and was conducted between September 2023 and January 2024. The study involved 64 participants, 50 of which were third-year dental students attending a pharmacology course focusing on antimicrobials in dentistry, comprising lectures and practical sessions without (noAPP group) or with (APP group) the assistance of a mobile application (https://www.dentalantibiotic.com; accessed on 4 November 2024). The other 14 participants were practicing dentists who decided to register and use the application. All registered users of the application were asked to take a feedback-survey consisting of three parts: First, the participants were asked to answer five questions using a 3-point Likert-type scale. Secondly, students graded the utility and applicability of the application in studying antimicrobials via a star rating system. Thirdly, the participants were given the opportunity to leave comments and suggestions. Undergraduate students voluntarily decided whether they wanted to use the application (n = 28) or not (n = 22) for preparing tests on antimicrobial use in the treatment of odontogenic infections. The test comprised 9 close-ended questions about proper pharmacological management of dental pain, odontogenic infections, and antibiotic prophylaxis, and each test was graded by a teaching assistant who was blinded from group assignment. Due to the voluntary nature of this study, the post hoc achieved power of the study was calculated, and a total of 50 participants were shown to be able to demonstrate a large difference (effect size: 1.20) in test scores between groups for a test power of 99% and an alpha probability of 0.05. Calculation was performed using a *t*-test for two independent means (statistical software package GPower 3.1).

### 4.2. Development of Mobile Application

The application covers four domains of antibiotic prescribing in clinical dental situations: (1) pulpal and periapical pain and swelling, (2) endocarditis prophylaxis, (3) antibiotic use in pregnancy, and (4) important information (e.g., information on when to refer to hospital and what each patient should be warned about). It was conceived based on a decision tree (Figure 1A) and written in Serbian, specifically the Cyrillic script (an official writing system in Serbia). Each student had to register prior to using the application. The application algorithm is based on the evidence-based dental antibiotic guidelines set out by the American Dental Association [2,5].

The application comprises two elements: the frontend UI/UX (User Interface/User Experience)-friendly app that is easily accessible for students and the backend server controlling access to available protocols and collecting voluntary, opt-in feedback from participants.

The frontend app is a PWA (progressive web application) available on the dentalantibiotic.com domain. It was written in Dart, using Flutter as the underlying technology to provide users with a beautiful, responsive interface and a consistent experience across multiple devices, namely, every device supporting up-to-date browsers (Firefox, Safari, Chrome, and Chromium-based browsers). The choice was made to distribute the app as a PWA because it considerably simplifies the distribution of the software—students are only required to navigate to the site, https://dentalantibiotic.com (accessed on 4 November 2024), in order to use all the features of the app.

The backend server is an ASP.NET Core MVC (Model–View–Controller) app deployed on a VPS (virtual private server) hosted by Oracle in their Zurich server region. The backend server abstracts the inner workings by providing a public API (application programming interface) to interact with client applications. ASP.NET Core is a modern multi-platform backend technology from Microsoft that eases the development of highly performant backend servers deployed on Windows, Linux, and MacOS. The app is written in C#. It controls the access of content by providing the login mechanism with token generation, enables administrators to invite new users via mail, and sends them automatic reports of opt-in feedbacks. Oracle provides two free-tier VPSs per account, and their integrity, security, and performance proved to satisfy our requirements without incurring additional costs.

### 4.3. Statistics

Data were managed by using GraphPad Prism software v.10. Results were expressed as mean score ± SD or percentage of students with correct answers on the pharmacological test. An unpaired *t*-test was used for the comparison of the two groups, while multiple linear regression was utilized to investigate whether there was a relationship between responses to feedback questions and clinical experience (undergraduate students or practicing dentists). A *p*-value of less than 0.05 was considered significant.

## 5. Conclusions

The dentalantibiotic.com application was developed to support rational antibiotic prescribing in an attempt to tackle misdiagnosis among inexperienced dentists and assist in undergraduates’ pharmacology learning. Students who utilized the application scored notably higher than those who did not, indicating that the integration of technology into learning can enhance knowledge retention and understanding of antimicrobial pharmacology in dental education. By offering interactive, practical decision-making scenarios, it bridged the gap between theoretical knowledge and real-world situations, helping to better prepare dental students for clinical work. The majority of participants considered it easy to use and efficient in facilitating the right antibiotic choice, also finding it useful for everyday decision-making. The use of this application aligns with the principles of personalized medicine and antibiotic stewardship, aiming to provide the most effective and safe care for patients and combat antibiotic resistance.

## Figures and Tables

**Table 1:** Relationship between feedback survey responses and participant status (undergraduates or practicing dentists). Data were obtained by multilinear regression.

Feedback Survey Questions	Estimate	Standard Error	95% CI (Asymptotic)	|t|	*p*-Value
Intercept	−0.32	1.10	−2.55 to 1.91	0.29	0.7711
Gender	0.19	0.25	−0.31 to 0.70	0.78	0.4413
Do you find the app easy to use?	0.20	0.61	−1.04 to 1.44	0.33	0.7409
Has the app facilitated the decision in the choice of AB compared to the literature that you usually used in decision-making?	−0.20	0.34	−0.89 to 0.48	0.61	0.5472
Is the app applicable to all patients?	0.11	0.17	−0.23 to 0.45	0.65	0.5195
Would you use the app in your everyday practice?	0.14	0.27	−0.40 to 0.69	0.53	0.6008
Has the app accelerated the decision in the choice of AB compared to the literature that you usually used in decision-making?	−0.001	0.29	−0.59 to 0.59	0.005	0.9957
Everyday utility (grade)	0.07	0.29	−0.53 to 0.68	0.25	0.8021
Assistance in learning (grade)	−0.05	0.35	−0.76 to 0.65	0.16	0.8755

## Data Availability

The data presented in this study are available on request from the corresponding author.
